# The Ōshū Fujiwara—An interdisciplinary study on the history, culture and medical assessment of the oldest known mummified human remains in Japan (late Heian, 12^th^ century AD)

**DOI:** 10.1371/journal.pone.0253693

**Published:** 2021-10-18

**Authors:** Sarah Rebecca Schmid, Michael Habicht, Patrick Eppenberger, Roger Seiler, Raji Steineck, Frank Rühli

**Affiliations:** 1 Institute of Asian and Oriental Studies, University of Zurich, Zurich, Zurich, Switzerland; 2 Institute of Evolutionary Medicine, University of Zurich, Zurich, Zurich, Switzerland; 3 Dep. of Archaeology, College of Humanities, Arts and Social Sciences, Flinders University, Adelaide, South Australia, Australia; University of Florence, ITALY

## Abstract

This study documents a rare case of mummified human remains from Japan, dating to the late Heian period, 12^th^ Century AD. The remains have only been scientifically investigated once in 1950 so far. The results of this investigation were translated, analyzed, and interpreted using methods of the 21^st^ century. The remains have been traditionally identified as the four ruling generations of the Ōshū Fujiwara clan, who built a cultural and economic center in Hiraizumi. Accordingly, this paper will first examine the historical and cultural significance of Hiraizumi and its ruling class before re-evaluating the findings of the 1950 investigation. This study is the first in the Western scientific literature to provide a comprehensive historical, cultural, and medical evaluation of these mummies.

## 1. Introduction and method

This study documents a rare case of mummified human remains in Japan, dating to the late Heian period, 12^th^ Century AD. The only scientific examination of the mummies took place in 1950 and was published as an investigation report (*Chūsonji to Fujiwara yondai*, 1950). Since the mummies themselves are currently inaccessible for scientific inquiries, this study is based primarily on the 1950 investigation report, a 1994 revised version of this investigation report (*Chūsonji goitai gakujutsu chōsa*, 1994), as well as a more recent reassessment of some of the x-rays from the 1950 investigation [1]. Since the overviews in the western scientific literature [2–4] are relatively limited, this study aims to provide access to so far untranslated and inaccessible parts of the original investigation report.

The paper is primarily divided into two parts: a first part providing historical and cultural background for both the location and the identity of the mummies, building on Heian and Kamakura period primary sources as well as on secondary sources, and a second part, which discusses the mummies themselves, based on the investigation reports and reassessments mentioned above. The paper’s medical part is heavily based on the original investigation reports, particularly the radiology report written by Taruzawa Sannosuke [5, 6] (*Chūsonji to Fujiwara yondai*, 1950, 67–82, and *Chūsonji goitai gakujutsu chōsa*, 1994, 100–147) with some adaptions to modern medical terminology. Indications about sources in this part are only made where important or necessary, such as comparisons to the re-evaluation [1].

## 2. Historical background

### 2.1. Late Heian period

Today, Hiraizumi 平泉 in Iwate Prefecture 岩手県 is a quiet, rural town with less than 8000 residents. During the late Heian period 平安時代 (794–1192), however, it was a major cultural, political, and economic center in the north of Japan, whose prime lasted for approximately one century.

In the capital of Heian (present-day Kyoto), the Fujiwara clan 藤原氏 *Fujiwara-shi* had wielded the de-facto political power during most of the Heian period through intermarriage between the Fujiwara daughters and the Japanese emperors. Towards the end of the Heian period, however, this relationship changed. The Fujiwara finally lost their grip on power at the end of the rule of Emperor Go-Sanjō 後三条天皇 (1034–1073), who abdicated in favor of his son, Emperor Shirakawa 白河天皇 (1053–1129). This move paved the way to the Insei system 院政, or cloistered rule system, in which the retired emperors and not the Fujiwara regents held the political power. This system remained in place until the beginning of the Kamakura period 鎌倉時代 (1185–1333). For an overview of the history of the Heian and Muromachi periods, see: [7].

Simultaneously, warrior elites (all of them descendants of the imperial household) steadily gained influence and power. The most powerful among these warrior elites were the Taira clan 平氏 *Heishi* and the Minamoto clan 源氏 *Genji*. After defeating the Minamoto during the 1160 Heiji Rebellion 平治の乱, the Taira replaced the Fujiwara as the most influential clan at the imperial court. However, only twenty years later, during the Genpei War 源平合戦 (1180–1185), the final victory in this conflict went to Minamoto no Yoritomo 源頼朝 (1147–1199), who subsequently founded the Kamakura Shogunate 鎌倉幕府 *Kamakura bakufu* (1185–1333). The victory of Minamoto no Yoritomo also marked the end for the Ōshū Fujiwara clan 奥州藤原氏in the north of Japan. Yoritomo effectually ended the rule of the Ōshū Fujiwara when he set out on the subjugation of Ōshū 奥州征伐 *Ōshū seibatsu* in 1189. With the violent death of the last heir of the Ōshū Fujiwara in the same year, Hiraizumi lost its status as an important center in the north, and the city gradually declined to a farming village.

### 2.2. The origins of the Ōshū Fujiwara clan

All mummies presented in the study belong to members of the Fujiwara clan. The establishment of the Ōshū Fujiwara clan is more difficult to trace than its end. Textual primary sources are scarce, as large parts of Hiraizumi were destroyed in 1189, and much of what remained got lost gradually over the following centuries. Many details about Hiraizumi during its prime are, therefore, unknown, and many of the sources still in existence were primarily compiled by the court or shogunate, thus giving rise to veracity and bias questions. Neither has archaeology so far succeeded in establishing concise geography of 12^th^-century Hiraizumi. Questions regarding the layout of the city and its surrounding temples and shrines remain unanswered. Even the status of the city and its leaders are not uncontested among researchers who hold varying opinions about the influence and independence of their rule; an overview of different positions can be found in: [8, 9].

Later sources such as the *Sonpi bunmyaku* 尊卑分脈 [10], compiled in the 14^th^ century, connected the Ōshū Fujiwara with the (widespread) Fujiwara clan in the capital by listing the Ōshū Fujiwara as descendants of Fujiwara no Hidesato 藤原秀郷 (10^th^ century), who had held the title *chinjufu shogun* 鎮守府将軍 (Supreme Commander of the Northern Defences). However, this connection cannot be fully verified. Historian Saito Toshio lists an entry from 1047 in the *Zō Kōfukuji ki* 造興福寺記, a document outlining repairs done on Kōfuku-ji 興福寺, one of the temples founded by the Fujiwara clan [11]. The entry contains a list of high-ranking Fujiwara nobility who made donations to rebuild Kōfuku-ji in 1047. One of the men listed was a man named Tsunekiyo 經淸 from Rokuoku 六奥, i.e., from Mutsunokuni 陸奥国 (Ōshū) or more specifically, from the six districts in the middle of Mutsunokuni known as Okurokugun 奥六郡 [12]. This Tsunekiyo is, according to Saito, Fujiwara no Tsunekiyo 藤原経清 (?-1062), the father of Fujiwara no Kiyohira 藤原清衡 (1056–1128), the first generation of the Ōshū Fujiwara.

The *Mutsu waki* 陸奥話記, a war chronicle written in the late 11^th^ century, also mentions Tsunekiyo as one of the casualties of the Zenkunen no eki 前九年の役 ("The Earlier, 9-Year Conflict", 1051–1063), a conflict between representatives of the imperial court in Kyoto and local elites. Tsunekiyo was beheaded together with members of the powerful local Abe clan 安倍氏 as punishment for their insurgence at the end of the war [13]. The war had mainly been fought between the Abe clan and another powerful local clan, the Kiyohara 清原氏, who was backed by Minamoto no Yoriyoshi 源頼義 (988–1075), the *chinjufu shogun*. It ended in the complete destruction of the Abe clan. The *Mutsu waki* further notes that Tsunekiyo was married to a daughter of Abe no Yoritoki 阿部頼時 (?-1057), then the head of the Abe clan, which might have been one of the reasons why he fought for the insurgent Abe side. The *Sonpi bunmyaku* lists Tsunekiyo as part of the local government of Mutsunokuni, though his exact role is not clear, and the researchers’ opinions vary.

### 2.3. Fujiwara no Kiyohira and the founding of Hiraizumi

It is generally accepted that Fujiwara no Kiyohira, the first generation of the Ōshū Fujiwara, was born to Fujiwara no Tsunekiyo and the above-mentioned daughter of Abe no Yoritoki in 1056. The *Ōshū gosannen ki* 奥州後三年記 tersely explains that after the death of Tsunekiyo in 1062, Kiyohira’s mother (whose name is unknown, a general issue with women of this period) remarried into the victorious Kiyohara clan, which had risen to become the most powerful local clan in Ōshū after the defeat of the Abe [13]. The marriage was likely a political move that aided in establishing Kiyohara authority in former Abe territories. His stepfather adopted Kiyohira, and henceforth he used the family name ’Kiyohara.’ Only much later, he would change his name back to Fujiwara, which was first recorded in 1091 [14].

Few details about Kiyohira’s life are known until the 1080s. A second larger conflict in Ōshū started in 1083, the *Gosannen no eki* 後三年の役 ("The Later, 3-Year Conflict"). It remained largely an internal conflict within the Kiyohara clan, ending in 1087 with all Kiyohara successors dead, except for Kiyohira himself. As the lone survivor, Kiyohira inherited the power his brothers had formerly held. However, according to Yiengpruksawan (1998, 60–62), Kiyohira was never awarded governorship over Ōshū. Instead, the governorship was given to a member of the Minamoto clan after the war. Despite this, Kiyohira rose to the top of the local elite and took on duties such as managing the estates (*shōen* 荘園) of various noblemen, levying taxes, and controlling trade. Ōshū produced some of the most coveted products in the capital: horses and gold. Rare trade goods from further north (for example, Hokkaido or the Asian continent) were also in high demand, and the local elites used all these desirable commodities as currency in exchange for influence in the capital. Though it is difficult to assert the exact status of these local leaders in the north, Yiengpruksawan [15] notes that "documentary evidence indicates that, at the very least, a semi-autonomous enclave existed in the Kitakami Basin that, seen as an ethnic bloc by all parties, was not entirely within the jurisdiction of the Japanese state". Other researchers, such as Takahashi Tomio, have suggested that Hiraizumi was de-facto independent [9, 14, 16].

Kiyohira moved to Hiraizumi after the *Gosannen no eki*, though it is not entirely clear when and how this move happened since no documentation from Hiraizumi itself survives. The *Azuma kagami* 吾妻鏡, the official history of the Kamakura bakufu, names the Kōhō 康保 era (964–968) as the time of the move (Masamune 1930, 230), but that is not possible. Since era names are often only one character apart, it is generally assumed that it must have been a mix-up with a similar, post-1087 era name—possibly Kahō 嘉保 1094–1096 or Kōwa 康和 1099–1104 [11].

The exact reason behind the choice of Hiraizumi as the seat of the Ōshū Fujiwara is unknown. Most researchers argue that it was both a strategical and a political decision [9]. The Kitakami river basin, where Hiraizumi is located, was connected to roads and waterways essential to the trade with the larger region in the north, including the continent. It was also directly south of the border of Okurokugun, the six districts that had been under the rule of the Abe clan before the *Zenkunen no yaku*, and not far from an old Abe stronghold at Koromo river. Therefore, it was further south than any of the lands over which the forerunners of the Fujiwara had ruled and simultaneously controlled the access to these lands from the direction of the capital.

With Kiyohira’s settling in Hiraizumi, a period of intense building started which lasted beyond his death almost until the final years of the Ōshū Fujiwara rule. The *Azuma kagami* [17] emphasizes that Chūson-ji 中尊寺, one of the still existing temples in Hiraizumi today, was built first. This temple, also known as Kanzan 関山, literally’ barrier mountain,’ lay directly on the main road leading further north, and Kiyohira made it the (spiritual) center of his domain.

Archaeological excavations have provided a clearer picture of Hiraizumi during the 12^th^ century in recent years, but many aspects remain unclear [18]. Though the *Azuma kagami* describes Chūson-ji and (the remains of) Hiraizumi, all original buildings of Chūson-ji except one have been destroyed, and the original layout of the temple is unclear. Yiengpruksawan [15] has presented a summary of what was known about Chûsonji’s layout until this point. Even though it is known that Kiyohira built a fortified mansion named *Hiraizumi no tachi* 平泉舘 in the city, and an excavation in 1990 revealed that this mansion had (almost certainly) been located in the area that is today known as *Yanagi no gosho* 柳之御所, other sections of the city, such as residential areas, have not been located yet.

If Kiyohira moved to Hiraizumi around 1090, as it is often assumed, he built and ruled there for approximately 40 years. Kiyohira died in 1128, at 72 years of age.

### 2.4. Fujiwara no Motohira

Kiyohira had several wives/consorts. For this reason, though Motohira’s mother is sometimes listed as a member of the Taira clan, it is not clear whose child Fujiwara no Motohira 藤原基衡 was. He was most likely born in 1105 as the second son of Kiyohira, with an age difference of approximately 15 years between him and his older half-brother Koretsune 惟常. According to the diary of Minamoto no Morotoki 源師時 (1077–1136), the *Chōshūki* 長秋記 [18], a conflict between Motohira and Koretsune broke out after Kiyohira’s death [19]. This conflict was ended by Motohira, who killed Koretsune and his family, thus effectively removing him and any competitors from the lineage.

Under Motohira’s rule, Hiraizumi expanded both in size and power. Motohira oversaw the expansion of Chūson-ji, as well as the building of Enryū-ji 圓隆寺 (located on the precincts of present-day Mōtsū-ji 毛越寺), and the founding of Kanjizaiō-in 観自在王院 by his wife (located right next to present-day Mōtsū-ji). Like his father, he continued with the management of *shōen*, expanded his territories, and funneled profits from noblemen’s estates into his own pockets, which brought him into conflict with the provincial government and the affected noblemen several times. At the same time, however, he was also in direct contact with the court in the capital, and a person of utmost authority and consequence in Ōshū itself, even though technically, the local governor appointed by the court was the one who ultimately ruled over the region, not Motohira. He died in 1157, at the approximate age of 52.

### 2.5. Fujiwara no Hidehira

Unlike his two predecessors, no textual sources point towards a struggle of succession in the case of the third generation of Ōshū, Fujiwara no Hidehira 藤原秀衡. He was most likely born in 1122 to Motohira and a woman of the Abe clan. With Hidehira, the power and influence of the Ōshū Fujiwara reached its zenith. Hidehira was appointed *chinjufu shogun* in 1170 and governor of Mutsu (Ōshū) in 1181 [20]. Such an appointment and elevation of status would normally have been unthinkable for someone of Hidehira’s origin, and his contemporaries received it with disbelief. Hidehira had grown powerful enough that the Heike clan sought to win Hidehira as an ally against the growing threat by Minamoto no Yoritomo, but Hidehira refused the alliance. Instead, he allowed Minamoto no Yoshitsune 源義経 (1159–1189), the younger half-brother of Yoritomo, to move to Hiraizumi as a teenager after he had grown up in a temple close to Kyoto. He would also continue to support Yoshitsune until the end of his life.

Hidehira continued to expand Hiraizumi itself, saw to the expansion of Mōtsūji as well as the building of Muryōkōin 無量光院, a temple constructed after the famous Byōdōin 平等院 which is still standing in Uji today. This also included the production of elaborate and expensive Buddhist artwork. For example, some descriptions can be found in the *Azuma kagami*, though most of Hiraizumi’s reported wealth has become the victim of fire and time [17]. Hidehira died on November 30^th^, 1187, which would make him around 65 years old at the time of his death.

### 2.6. Fujiwara no Yasuhira

Fujiwara no Yasuhira 藤原泰衡was the last head of the Ōshū Fujiwara. Due to his short period of rule, he is sometimes not counted by historians, making the lineage three generations instead of four. Yasuhira was born as the second son of Hidehira in 1155. He had an older brother, but while Yasuhira was born to Hidehira’s legal wife, a daughter of Fujiwara no Motonari 藤原基成, his older brother was not. Most likely, Yasuhira became the successor of Hidehira for that very reason.

Due to Minamoto no Yoshitsune’s popularity as a tragic hero in literature and the arts, Yasuhira is primarily remembered as the man responsible for his death. Yasuhira’s father, Hidehira, had consistently supported Yoshitsune and reportedly requested before his death that Yasuhira continue to do so [20]. At first, Yasuhira apparently complied but then folded to continued pressure by Yoritomo, who had come to see his half-brother and former ally as a threat. Yasuhira attacked and besieged Yoshitsune, which lead to Yoshitsune’s suicide at thirty years of age.

When Yasuhira presented Yoshitsune’s severed head to Yoritomo, he did not receive the perhaps hoped-for peace. Instead, Yoritomo headed with an army towards Hiraizumi. According to the *Azuma kagami* [17], Yasuhira chose to flee Hiraizumi after setting fire to his city instead of fighting. During his flight north, however, he was beheaded by one of his vassals. On October 14th, 1189, he died after only two years of rule over Hiraizumi around age 34. With his death, the rule of the Ōshū Fujiwara and the golden age at Hiraizumi came to a definite end.

## 3. Konjiki-dō, the “Golden Hall” in Chūson-ji

Although it was significantly larger during the rule of the Ōshū Fujiwara, Chūson-ji today consists of seventeen temple buildings spread out over a larger area (https://en.wikipedia.org/wiki/Ch%C5%ABson-ji). The layout of the original temple complex is unknown, but it was intended to represent the Western Pure Land and a symbol for peace in the realm that the Ōshū Fujiwara ruled [15]. The veneration of the Buddha Amitābha (Jap. Amida nyorai 阿弥陀如来) spread among the elite during the Heian period, and the practice of building and supporting so-called Amida Halls (阿弥陀堂 Amida-dō) became popular. Amida nyorai, it was believed, would deliver his devotees to the Pure Land in their moment of death, where they would be reborn and eventually achieve enlightenment through the instruction of Amida nyorai himself.

Chūson-ji stands in this tradition, though the main icon of the temple today is Siddhārtha Gautama (Jap. Shaka nyorai 釈迦如来). The Konjiki-dō 金色堂, however, the last surviving original structure from the period of the Fujiwara rule, completed in 1124, is one of the aforementioned Amida Halls that have Amida nyorai as its main icon. The Konjiki-dō is a small building that originally stood on the grounds of Chūson-ji uncovered. It is almost entirely covered in gold leaf (hence the name "Golden Hall"). The pillars and dais inside the building are covered with elaborate decorations made from mother-of-pearl inlays, metal, lacquer, and other precious materials. During the restoration work in the 1960s, it became clear just how elaborate and unique the techniques used in the Konjiki-dō were, especially the shell inlays and lacquer- and metalwork bodhisattva images on the pillars, as they were difficult to recreate even with modern techniques. A good overview of the restoration works is the film "*Yomigaeru Konjiki-dō*" by the *Nichiei kagaku eiga seisakusho* 日映科学映画製作所 (1970) [21], and an English introduction to Chūson-ji can be found on Youtube [22].

Though the Konjiki-dō has a basic square shape, each side measuring a maximum of 5.5 meters and has a total height of 8 meters, the craftsmanship level packed into this small hall is considerable.

Most of the interior of the Konjiki-dō is taken up by three daises serving as altars for three central icons. All three central icons are seated figures of Buddha Amitābha, surrounded by the Bodhisattvas Avalokiteśvara (Jap. Kannon bosatsu 観音菩薩) and Mahasthamaprapta (Jap. Seishi bosatsu 勢至菩薩), who commonly accompany Amitābha in Pure Land Buddhist iconography. The other statues on the altars are six Kṣitigarbha (Jap. Jizō bosatsu 地蔵菩薩) statues, as well as Dhṛtarāṣṭra (Jap. Jikoku-ten 持国天) and Viruudhaka (Jap. Zōchō-ten 増長天). This combination of Buddhist deities is, according to Chūson-ji itself, unique. A detailed description of the interior of the Konjiki-dō and the techniques and the iconography used can be found in Yiengpruksawan [15] and the catalog published by Chūson-ji [23].

The mummified bodies of the Ōshū Fujiwara rulers have been placed beneath these three altars of Amida nyorai. Each dais contains one body, except for one, which contains a body and another mummified head, with the body missing. All three bodies are placed in caskets aligned north-south, in accord with the belief that Gautama Buddha chose this position (with the head facing north) when he passed away. The Konjiki-dō itself is aligned east-west, facing Amida’s “Pure Land in the West.” The mummified head was placed in a separate wooden bucket with a lid. According to Yiengpruksawan [15], there are no mentions of the mummified bodies before the 17^th^ century, but from 1624 onwards, the bodies have been exhumed at least four times before the first scientific investigation in 1950. Therefore, it is unclear when, how, and why the mortal remains were placed in the Konjiki-dō. Since the restorations of the hall revealed that the three daises had not been built at the same time, it can be assumed that those were explicitly created to enshrine the bodies of the Fujiwara rulers.

**Fig 1:**
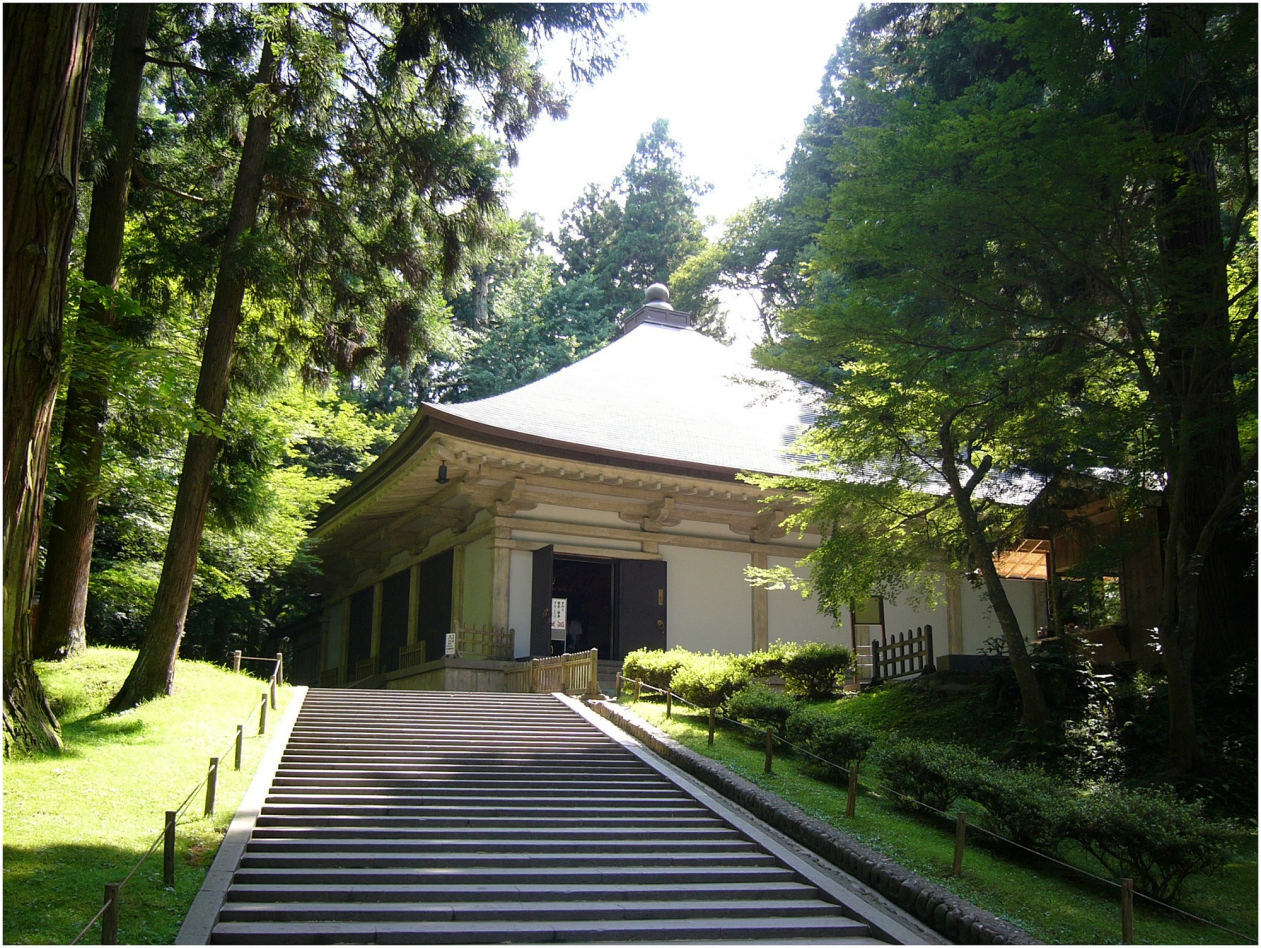
External protection shelter made of concrete (20^th^ Cent.) protecting the golden shrine inside. https://upload.wikimedia.org/wikipedia/commons/2/2e/Konjikido-Ooido.jpg. 竹麦魚(Searobin) (https://commons.wikimedia.org/wiki/File:Konjikido-Ooido.jpg), "Konjikido-Ooido ", https://creativecommons.org/licenses/by-sa/3.0/legalcode.

In 1288, a larger wooden building was built surrounding the Konjiki-dō to protect it from the elements. This is how it remained for centuries until the outer building and the Konjiki-dō were heavily damaged in a storm. The Konjiki-dō was subsequently restored between 1962 and 1968 and moved into a modern building made of concrete that encloses and protects the Golden Hall (Fig 1). Chūson-ji and the Konjiki-dō are now a part of the Hiraizumi World Heritage Site, declared by UNESCO in 2011. Incidentally, the Konjiki-dō had also been among Japan’s first structures to be designated a National Treasure.

## 4. Mummification in Japan

Japan does not have a general tradition of mummification. East Asia’s climate does not favor natural mummification in the soil, making extensive mummification practices, more commonly known from drier climates, impractical. The mummies in the Konjiki-dō are not in soil but still exposed to the humid climate to a certain extent: The climate in Hiraizumi, for example, is characterized by relatively low temperatures and high humidity levels which do not make it an ideal place for mummification. According to data from the Japan Meteorological Agency, Morioka (the closest larger city from Hiraizumi) had an annual mean temperature of 10.2 C° and relative humidity of 74% between 1981 and 2010 [24]. Recent paleoclimatological reconstructions for central Japan [25] show climatic variations since the medieval period comparable to global temperature and East Asian precipitation models, implying a somewhat drier climate during the medieval climate anomaly from about AD 900 to 1200 [26], followed by a more humid climate during the Little Ice Age from the 16^th^ to the 19^th^ centuries [25, 27]. Nevertheless, such climatic conditions are still quite far from facilitating natural mummification even with slightly less humidity.

Despite the absence of a general tradition of mummification, the practice of *sokushinbutsu* 即身仏 (literally: ’becoming a Buddha in this very body’) produced several examples of mummified bodies, some of which have survived to the present day. Kūkai 空海 (774–835) was one of the first to whom such a practice was related. He was said to have entered a state of intense meditation in a cave on Mount Koya 高野山 in Wakayama Prefecture 和歌山県 instead of dying in 835, and legend holds he remains there to this day, awaiting the arrival of the future Buddha Maitreya (Jap. Miroku bosatsu 弥勒菩薩). The story, referred to as *Kūkai nyūtei densetsu* 空海入定伝説, was frequently retold during the Heian period in works such as the *Eiga monogatari* 栄華物語, the *Konjaku monogatari* 今昔物語 as well as specifically Buddhist texts. During the same time, several instances of monks and noblemen either becoming mummies or failing to decompose for a significant period after death were recorded: for details, refer to the timeline in [28] or a comprehensive list of Japanese Buddhist monk mummies in [29]. The religious situation at the time was therefore not unwelcoming to instances of physical preservation after death, at least in the imperial domain. Whether such concepts also influenced the reasoning of the Ōshū Fujiwara, however, needs further research.

Also, most of the practitioners referred to as *sokushinbutsu* are not from the Heian or even Kamakura period, but from the Edo period 江戸時代 (1603–1868). Most of these practitioners were *shugensō* 修験僧 (monks) from Dewasanzan 出羽三山, and among the still existing *sokushinbutsu*, examples from Niigata and the Tohoku region are the most numerous [29, 30]. These practitioners often practiced *mokujiki* 木食, meaning they did not eat certain types of cereals (usually five or ten types) and stopped eating entirely before their deaths, though the details of what they did or did not eat are different in every case. This extensive preparation differs significantly from the appearance of the remains of the Ōshū Fujiwara, who show no sign of ascetic practice and who were well-fed at the time of their death.

The geographic overlap between the domain of the Ōshū Fujiwara and the *sokushinbutsu* does not exclude that the Fujiwara might have been influenced by similar ideas, albeit the temporal distance makes this unlikely. The authors of the 1950 examination report [5] discussed whether the mummification occurred naturally or unnaturally, but they were ultimately unable to resolve their disagreements. Hanihara [31] stated that the bodies were mummified naturally and that their skin condition was indicative of natural mummification, and no traces of the artificial removal of organs could be found. Still, he questioned that all three bodies could mummify while left entirely untouched. His final suggestion was that the drying of the bodies was facilitated somehow—this would imply, however, that the mummies were at least partially "artificially" mummified, i.e., that the process of decomposition was somehow manipulated. However, so far, no actual supporting or contradicting evidence has been reported.

Hanihara (1996) also suggested that the Fujiwara knew a custom called ’*mogari*’ 殯, usually practiced for an emperor’s funeral. *Mogari* is a custom where one does not immediately bury the body of a deceased person but enshrines it in a so-called *mogari* shrine to mourn the person’s loss, perform rites and wait for the ultimate decomposition of the body. In earlier times, this custom was also practiced for other members of the nobility. Under ideal conditions, such a custom might have facilitated natural mummification. Here again, however, there is no evidence linking this custom to the Ōshū Fujiwara.

Two participants of the 1950 investigation, Furuhata Tanemoto, and Mori Kahei, cited Mamiya Rinzō’s 間宮林蔵 (1775 or 1780–1844) 1808–10 expedition account to Sakhalin, *Kita Ezō zusetsu* 北蝦夷図説 (published in 1855), as well as Kondō Morishige’s 近藤守重 (1771–1829) sketches from *Henyō bunkai zukō* 辺要分界図号 (written before 1804?) reporting that the Ainu アイヌ of Sakhalin had a tradition of artificial mummification [6]. They premised this to suggest the Fujiwara had used a similar technique to that of the Ainu. In the Ainu tradition, the dead bodies were eviscerated and dried in the sun before burial. Although such a mummification practice may have existed on Sakhalin in the 19^th^ century, this was not necessarily the case in the 12^th^ century. Hasebu Nobuto rejected this theory arguing that the Ainu of Hokkaido, which is closer to Tohoku, did not know such a tradition, and therefore the question of how the Fujiwara would have acquired knowledge of such a technique was unclear [5]. However, it would not be entirely impossible since the trade connections of Hiraizumi were significant and extensive. Hasebu, along with Suzuki Hisashi, also rejected the idea that the bodies had been artificially mummified, arguing that no traces indicated that organs had been deliberately removed. The ideological background of the preservation of corpses was also discussed at the 1984 "Tohoku Culture Symposium in Hiraizumi" ("東北文化シンポジウム 平泉"), but no clear conclusion was found, as summarised in Takahashi [32].

## 5. The Ōshū Fujiwara and ethnicity

According to Ōishi [8], a significant part of Hiraizumi-centric research undertaken before the Second World War was concerned with anthropological questions, such as whether the Ōshū Fujiwara were entirely ’Japanese’ (i.e., had blood ties to the Fujiwara in the capital, see chapter 3.2) or if they were part of the ’local’ Emishi 蝦夷 or Ainu population. How the importance of the culture developed in Hiraizumi should be viewed in the larger context of Japanese history was also linked to ethnicity—was it copied from the culture in the capital, or was it ’barbaric’? Needless to say, such a line of research—partly rooted in racial theories, with the Emishi and Ainu considered inferior—can easily veer into problematic territory. Also, there is no indication that the Emishi ever were a clearly defined, separate ethnicity since contemporary sources lack such mention. However, such conceptions did not end with the war but remained resonating in the post-war era. As Hopson states, "… race, history, and their relationships to national identity were front and center in the minds of the small group of scholars who clustered around the coffins of the Fujiwara men in the spring of 1950" [9].

This must be kept in mind when reading and analyzing the examination reports from 1950, where the topic of ethnicity is extensively discussed by several of the contributors (see, e.g., chapter 5). Hanihara Kazurō, who contributed to the second publication of the investigation records in 1994, also commented on the subject. As a physical anthropologist, he was particularly interested in craniometry and added his research on craniometric data to the 1994 report [6] (Table 1). The objective for measuring their skulls was to provide insight into the origin of the Ōshū Fujiwara. His research stated that the cranial measurements positioned them closer to modern-day inhabitants of Kyoto than of Tohoku. However, ultimately, this must be considered cautiously, in part because of the investigated specimens but also because of longstanding preconceptions toward the people of Tohoku and Hokkaido. Ethnicity is not merely a scientific question, but a matter of cultural prejudice and, as Hopson has mentioned, national identity and historiography. The broader implications of categorizing the Ōshū Fujiwara as ’Japanese,’ ’Emishi’ or ’Ainu’ cannot and thus should not be disregarded in scientific research.

**Table 1:** The cranial measurements based on Hanihara [6].

Measured sizes	Kiyohira	Motohira	Hidehira	Yasuhira
Cranium maximum length	180	187	190	186
Cranium maximum width	144	152	148	147
Cephalic index	80.0	81.3	77.9	79.0
Basion-bregma height	133	142	-	142
Length-height index	73.2	75.9	-	76.3
Breadth-height index	92.4	93.4	-	96.6
Malar arch	138	135	139	138
Face height	118	129	137	116
Upper face height	71	79	77	67
Facial index	85.5	95.6	98.6	84.1
Upper face index	51.5	58.5	55.4	48.6
Nasal height	59	59	57	51
Nasal width	26	25	26	-
Nasal index	44.1	43.2	46.1	-
Orbital height	-	39	38	37
Orbital width	-	46	44	46
Orbital index	-	84.8	86.4	80.4

An approach potentially providing a broader picture of the origin of the Ōshū Fujiwara—if only through the patrilineal lineage—is Haplogroup (Jap. ハプログループ) population genetics. Since DNA analysis was unavailable during the 1950s examination, no suitable samples seem to have ever been taken or tested. On the other hand, some of the Fujiwara descendants must have been tested, as they are listed in the genetic database Y-DNA-12 (https://www.familytreedna.com/public/o3?iframe=yresults). The Fujiwara shall be O1b2a1a1 (CTS11723 tested or predicted), while the family line of the Tennō is D1b1a2 (IMS-JST022457) (https://en.wikipedia.org/wiki/Haplogroup_D-M174). However, even at present, a state-of-the-art aDNA analysis of the Fujiwara mummies may not provide additional evidence in this respect, provided they were members of the same male family line and would share a Y-chromosomal haplogroup. The mortal remains of the mothers and wives of the Fujiwara are not preserved, nor are the actual mothers confirmed in all cases, considering that all the clan members were practicing polygamy and that nominal adoption (i.e., the adoption of the son of one of the concubines by the legal wife) was widespread [33].

The result of the blood type testing done on the mummies during the 1950s examination:

- Kiyohira AB
- Motohira A (Motohira and Hidehira might be reversed.)
- Hidehira AB
- Yasuhira B

## 6. The mummies

**Fig 2:**
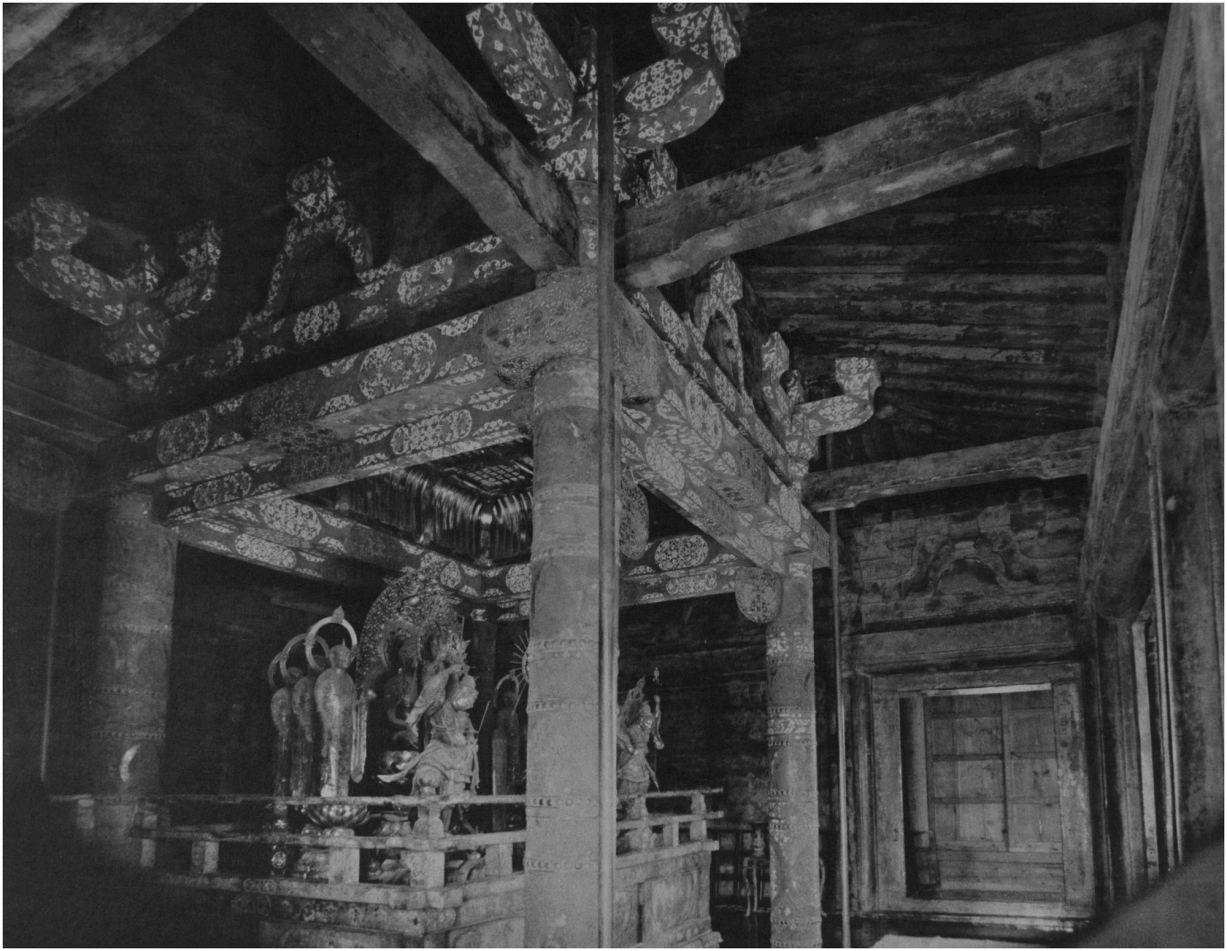
Central altar of the shrine, photographed in 1915. https://upload.wikimedia.org/wikipedia/commons/3/3e/Interior_of_Konjikido%2C_Chusonji_%2863%29.jpg. Imperial Japanese Commission to the Panama-Pacific International Exposition (https://commons.wikimedia.org/wiki/File:Interior_of_Konjikido,_Chusonji_(63).jpg), "Interior of Konjikido, Chusonji (63) ", https://commons.wikimedia.org/wiki/Template:PD-Japan.

For our radiological follow-up, we had available high-resolution digital scans from the original radiographs of the 1950 examination [5], republished in 2017 [1] as Figs 1–7. We evaluated both the highly detailed original Japanese reports from 1950 and the English summary reports from 2017 and consolidated them with our radiological re-evaluation.

### 6.1. The mummy of Fujiwara no Kiyohira

The identification of the four mummies is not beyond any doubt. However, it is generally accepted that Fujiwara no Kiyohira, the founder of the dynasty and patron of Chūson-ji, is the one enshrined below the central altar since it was probably the first constructed altar (Fig 2). The original examination report provides the following findings of his mummy [5]:

#### 6.1.1. Cranial findings

The cephalic (length-breadth) index of Kiyohira is 80.30 (brachycephalic, but only slightly above mesocephalic), Tamura et al. [1] came to the same conclusion.

The sagittal suture is relatively distinct, the lambdoid suture fine and convoluted (cloud-shaped), the coronal suture is visible on the left but not on the right. A prominent exostosis protrudes from the inion (external occipital protuberance), likely a sign of chronic enthesitis, typically caused by strenuous use of the trapezius and semispinalis capitis muscles inserting in this area [34]. The median meningeal artery’s sulcus and the diploic veins’ impressions do not exhibit any notable alterations. The alveolar processes of the upper and lower jaws are atrophied with pronounced exposure of the dental roots. On the left side of the mandible, the canine and the second incisor show significant wear. On the mandible’s right side, the dental wear is particularly pronounced for the first molar, the second and the first premolars, the canine, and the second incisor. The occlusal surfaces and the margins are flat or concave. This is most likely a sign of old age. Almost all the mandibular dental remnants (left second, first molars, second, first premolars, canines, second incisors) have cavities.

#### 6.1.2. Post-cranial findings

The collarbones are short and thick, like those of a laborer (i.e., physical work; this observation fully concurs with the life of Kiyohira as a warrior). It can be assumed that both collarbones have been broken at some point during his lifetime. The rib cartilage shows signs of parasternal osseous healing; the episternum and the sternum’s body also show signs of osseous healing. The lumbar spine has spondylosis deformans.

The right femur shows periosteal osseous deposits; the left femur, the left tibia, the left fibula, the left proximal radius, and the left ulna show distinct signs of skeletal atrophy, including loss of volume. There are no marked signs of skeletal atrophy on the right side. The ilium also demonstrates greater radiolucency on the left side than the right side.

#### 6.1.3. Interpretation

Looking at the investigation findings, it is possible that a right-sided cerebral hemorrhage or cerebral embolism developed, leading to contralateral hemiplegia on the left side, causing disuse-atrophy. Consequently, the blood circulation might even have been compromised on the paralyzed side, worsening bone atrophy due to insufficient supply. Tamura et al. [1] considered that hemiplegia-related osteoporosis was the most likely explanation and discussed cerebral hemorrhage (or a similar condition) as a possible cause of death. Further investigations would be needed to clarify this [35]; however, a lethal external injury would be expected to affect both sides and lead to death before bone atrophy can develop. Judging from the X-rays (applying diagnostic criteria such as bone maturation and the extent of degenerative alterations), Kiyohira is likely the oldest among the four bodies, estimated over 70 years of age [1]. This corresponds to Kiyohira’s reported age at death of 72.

### 6.2. The mummy of Fujiwara no Motohira

The north-western altar (the right dais) is considered the resting place of Fujiwara no Motohira. However, there is a possibility that the position of the second and the third Fujiwara may be interchanged. Since there are no early records of the mummies’ placement or their identities, the identification commonly accepted today is the one reported by the temple.

The original examination report provides the following observations regarding the mummy of Fujiwara no Motohira [5]:

#### 6.2.1. Cranial findings

The cranial index is 81.09% (brachycephalic). The lambdoid suture is partially fused and only intermittently distinguishable; the sagittal suture only appears straight due to the projection’s angle. The coronal suture is not visible.

The median meningeal artery’s sulcus and the impressions of the diploic veins are very prominent. Pronounced diploic veins can be caused by elevated intracranial pressure. Intracranial pressure is associated with several health conditions, such as brain tumors or apoplexy. However, the exact cause could not be determined in 1950, and the authors suggested further research. A slight exostosis protrudes from the inion (external occipital protuberance). Again, likely a sign of enthesitis probably caused by strenuous use of the trapezius and semispinalis capitis muscles inserting in this area [34], potentially related to either strict manners (such as being forced to sit or stand in certain rigid positions for an extended period), or use of body armor (helmet), the practice of swordsmanship, and practice of archery. The mandible shows pronounced atrophy of the alveolar process, pronounced tooth root exposure, and a general rarefaction of bone trabecula. The first and third molars on the left side of the mandible have fallen out during his lifetime, whereby the first molar seems to have fallen out relatively early in life. The second molar is still present. The root of this tooth does not split but is a single root. The occlusal surfaces and the marginal edges of almost all teeth of the left mandible (second, first premolar, canine, lateral incisor) are worn and flat or concave. These dental abrasions, along with alveolar bone atrophy, are a sign of old age.

#### 6.2.2. Post-cranial findings

There is no radiological evidence of the heart or the lungs being preserved in the thoracic cavity. There are almost no intestinal remains discernible within the pleuroperitoneal cavity. On the other hand, thoracic X-rays reveal several bone fragments and human teeth, as well as a patella beneath the right side of the spine at the level of the third and fourth lumbar vertebra. There are fractures of the left third and fourth ribs and the right second, third, and twelfth ribs. Further, numerous beads, two pieces of horn, one piece of metal, an object looking like a wrinkled gold leaf, and two or three gold rings were discovered. The ileo-sacral region shows signs of spina bifida occulta (i.e., probably without disabling effects). There is an ornament in the left and central parts of the pelvis, respectively. Two *yōraku* 瓔珞 (Buddhist hanging ornaments) were discovered on the left side, along with several pieces of human teeth and bones and what seems to be a rectangular plate made of horn.

#### 6.2.3. Interpretation

It is unclear whether the items mentioned above were introduced after death, moved there by mice, or fell into the cavity from outside of the body. Tamura et al. [1] alleged that rodents might have damaged the mummy in the past, but this cannot be confirmed based on the currently available data. The radiographic evaluation of the cranial sutures, the spine, and the mandible, suggests that this is the youngest of the three bodies, with age at death of approximately 50 to 60 years. This is also supported by Tamura et al. [1].

### 6.3. The mummy of Fujiwara no Hidehira

Following the standard theory, Fujiwara no Hidehira rests under the altar to the left (south-eastern altar). A cluster analysis of the cranial proportions by Hanihara [6] among the four Fujiwara showed that Kiyohira and his son Motohira cluster closer to each other, likewise does the other pair, Hidehira and Yasuhira. It has (consequently) been suggested that both are father-son pairs. However, the missing mothers are an unknown factor, and Motohira and Hidehira are also a father-son pair.

The original investigation report supplies the following findings for Fujiwara no Hidehira’s mummy [5]:

#### 6.3.1. Cranial findings

The occipital bone features a separate interparietal bone, and an outer entheseal protrusion is visible as a slight flat bulge at the inion, under which an additional adjacent extracranial calcified structure can be seen. In contrast to the comparable protrusion of the inion of the first two subjects (Motohira), here, particularly the muscular portions preceding the actual enthesis seem to be (partially) calcified. It can be assumed that these findings, besides an individual predisposition, can also be attributed to similar excessive strain on the respective muscles (i.e., sword fighting, wearing body armor, or strict contemporary manners) [34]. No further abnormalities, e.g., the impressions of the diploic veins or the median meningeal artery’s sulcus, can be identified in the cranial X-rays.

#### 6.3.2. Post-cranial findings

Both scapulae exhibit large entheseal protrusions on their medial and lateral margins, indicating that the inserting muscles (teres major, teres minor, serratus anterior, triceps brachii) were well developed, likely due to intense use of these muscles (e.g., archery). The lungs’ remains cannot be identified in the thoracic cavity; however, the diaphragm and parts of the heart and the aorta are still discernible in situ. In the upper thoracic spine, the intervertebral spaces are shadowy, complicating the vertebral bodies’ delimitation. The vertebral bodies below the sixth thoracic vertebra, including the entire lumbar spine, are sclerotic, exhibit large osteophytes, and are partially fused by bony bridges. Further ossification of the anterior longitudinal ligament of the thoracic spine can be seen. Overall, the thoracic radiographs are challenging to assess due to irregular shading. Remains of abdominal organs such as the spleen, liver, stomach, intestines, or kidneys cannot be identified. It is unclear whether these organs have been removed or not, but the abdomen’s left side seems to have been opened. The edge of the ischium shows a distinct spine-shaped protrusion. The femur’s posterior edge (i.e., the linea aspera) is unusually prominent and unevenly protruded. There is a partial ossification of the patellar tendon at the lower edge of the left patella, which can also be seen on the right side. The calcaneus displays a beak-shaped protrusion on both sides, probably due to intensive strain on the Achilles tendons.

#### 6.3.3. Foreign objects

Inside the pelvis and along the pleuroperitoneal cavity, several human teeth can be distinguished along with two (presumably ancient) coins. Laterally of the right ileum, two other objects can be identified, possibly two further coins. Another coin was found within the musculature of the right thigh. In the 1994 publication [6], however, no coins are mentioned, only a metal ring. A square object measuring one centimeter made of gold was found beneath the right shoulder and two golden spheres with a diameter of approximately 5 mm, and a golden ring with a diameter of approximately 4 mm.

#### 6.3.4. Interpretation

The alterations of the spine are indicative of diffuse idiopathic skeletal hyperostosis (DISH), also known as Forestier’s disease, characterized by bony proliferation of the spine at sites with tendinous and ligamentous insertions typically in older people.

These findings imply a considerable stiffness of the vertebral column already during Hidehira’s lifetime, most likely related to old age and possibly intense martial arts training. The femora’s entheseal protrusions were likely caused by intensive use of the adductors, probably related to (horse)-riding. Riding, among other things, may also have caused the alterations of the calcaneus. It was impossible to determine whether the identified foreign objects were inserted after death, moved there by mice, or had fallen into the thoracic cavity from outside of the body after decay. These objects are, therefore, probably still in the body for the time being. From the X-ray images, it was concluded that it is likely that Hidehira almost reached the high age of Kiyohira. Tamura and colleagues support this, estimating his age at death to about 70 [1].

### 6.4. The mummified Head of Fujiwara no Yasuhira

Only Yasuhira’s severed and mutilated head is preserved. The temple lore of Chûson-ji traditionally identified the head as that of Tadahira 忠衡, the younger brother of Yasuhira. The researchers suggested the new identification in 1950 because the historical record claims Yasuhira was beheaded and an iron nail stuck through his head as punishment. The injuries on the preserved head are consistent with this claim, and the head features the following injuries:

- Cuts across the face and cranium
- A hole the size of a fingertip on the forehead
- A slightly smaller round hole at the back of the head

The 1994 final report on the mummies gives insight into the anthropology and pathology of Yasuhira’s head [6]:

Yasuhira’s head is classified as brachycephalic, with a cephalic index of 78.19. The sagittal suture is wave-shaped in the front. A gap in the lambdoid suture is particularly well visible, showing a filamentous suture. All visible cranial sutures show signs of incomplete fusion. Judging from the sutures alone, the head of Yasuhira looks closer to that of a 25-year-old man. However, looking at the weak cloud-shaped ossification of the surrounding bone, it may be closer to that of a 35-year-old individual. According to the state and growth of the teeth, his age at death is set to the early thirties. A complete dental status is given in Table 2.

**Table 2:** Dental status of Fujiwara no Yasuhira’s mummified head.

**Left mandible**	**Right mandible**
First incisor: some sign of tooth attrition	First incisor: some sign of tooth attrition
Second incisor: No sign of tooth attrition.	Second incisor: very light sign of tooth attrition.
Canine: Sings of tooth attrition, concavity of the chewing surface	Canine: light sing of tooth attrition, otherwise nothing specific
First premolar: nothing specific	First premolar: nothing specific
Second premolar: nothing specific	Second premolar: nothing specific
First molar: two roots, full growth of the root, nothing specific.	First molar: two roots, full growth of the root, nothing specific (image unclear).
Second molar: two roots, full growth of the root, nothing specific.	Second molar: two roots, full growth of the root, nothing specific (image unclear).
Third molar: Two roots, apex of the mesial root is unclear, but it is assumed it is complete.	Third molar: two roots, full growth (image unclear)
**Left maxilla**	**Right maxilla**
First incisor: almost no sign of tooth attrition	The first incisor has fallen out. Due to traces on the lower incisors likely after death or shortly before death.
Second incisor: Damage, half of the tooth is broken off (the other half remains in the alveolar)	Second incisor: centrifugal distortion?
Canine: nothing specific	Canine, first and second premolar: nothing specific
First premolar: nothing specific	
First molar: nothing specific	First molar: fallen out, likely during lifetime
Second molar: nothing specific	Second molar: three mesial roots
Third molar: nothing specific	Third molar: unclear, but likely nothing specific

The glabella protrusion is low and smooth, the concavity of the nose’s root is shallow, and the nasal bone low. A sword wound of about 30 mm depth is found on the left parietal bone. The wound is covered by soft tissue and cannot be seen from the outside. A bit further up and to the medial side is a laceration of the skin (or soft tissue) likely caused by a blade during his lifetime. There is an oval-shaped detachment of bone (around 40mm in diameter) in the right occipital region. On the left side, there is also an oval hole (17x10mm), likely caused by a sword. On the forehead’s left side is a nearly round hole, which measures 16x18.5mm on the external lamina and 22x21mm on the internal lamina. Light fissures run downwards from the hole on the external lamina. In direct opposition to the hole on the front is a further hole on the back, which is the smallest of the three holes with 11x13mm. The hole on the lamina interna is smaller than the one on the lamina externa. Due to the type of breakage, a spike or nail likely caused the damage. There is a laceration on the left mastoid process. The fourth cervical spine shows a sharp slanted cut. Based on the injuries, Yasuhira was likely caught, beheaded, and pierced by a nail during or shortly after the battle. This corresponds to the passage in the *Azuma kagami* [16], which related that Yasuhira was indeed beheaded, and his skull was pierced by an iron nail measuring eight *sun* (approx. 24 cm). A nail of that length would easily manage to pierce through the front and back of the head since Yasuhira’s cranium has a maximum length of 18,6 cm. There were no further abnormal radiological findings of the osseous structures of the facial skull. In the skull vault, the groove for the middle meningeal artery is well distinguishable. However, the sella turcica shows a somewhat rare pattern sometimes seen in young people: The anterior clinoid process and the dorsum of the sella turcica show osseous conglutination, which resembles a beehive pattern in the lateral projection. The anterior clinoid process and the dorsum sellae were initially connected by connective tissue, and part of this connective tissue seems to be calcified. However, it is unclear whether or not this was related to a dysfunction of the pituitary gland.

Yasuhira’s head, in the opinion of Hanihara [31], looks more like an adipocere corpse than a mummy, which makes it stand apart from the other three mummies. According to him, this results from natural dehydration and saponification of the fat under the skin. The *Azuma kagami* mentioned that the head was transported after Yasuhira’s demise, meaning that it was most likely placed in salt or another preservative to prevent the flesh’s rotting. The actual mummification must have occurred after this preventative measure through moisture absorption and drying.

## 7. Discussion

Most of the intriguing questions about the four mummies remain unanswered: identification, cultural significance, and method of mummification.

The overall verdict is that it is highly probable that the identification of the four generations of the Ōshū Fujiwara is correct as proposed in the examination reports. Since the ages determined by the researchers closely correspond with their traditionally reported ages at death, there is no indication that this identification should be inaccurate. Also, the pathological and degenerative alterations identified on the bodies correspond to the men’s reported lifestyles. For Yasuhira’s head, in particular, the injuries correspond to those described in the *Azuma kagami*. However, there is no scientific evidence capable of identifying them beyond any doubt. To accomplish that, most likely, a genetic analysis of their familial relations would be required.

Unfortunately, the study of contemporary sources could not elucidate the motives for placing the mummies in the Konjiki-dō and what cultural significance this placement held, both for those placed within the dais and those caring for the Konjiki-dō and visiting it. Further study is necessary for this particular field, and it is also the most easily accomplishable kind of research at present. Whether the bodies’ mummification occurred naturally or artificially has remained disputed among researchers, along with the cultural background and ideology of potential artificial mummification practice. One of the reasons for this determination’s difficulty is the damage that the bodies have accumulated over the centuries, making it difficult to determine in which condition the mummies were initially placed into the Konjiki-dō. Besides, Yiengpruksawan mentioned that the mummies were exhumed several times after the seventeenth century, which might have altered the mummies’ condition. According to an old documentary in possession of Chūson-ji, the remains had also been exhumed in 1931 and the coffins lined with asbestos. The asbestos has been removed during the 1950 investigation, but the impact on the mummies of both the asbestos and its removal remains unclear.

The study cannot answer the question on the present condition of the Ōshū Fujiwara mummies. A re-inspection for conservation purposes might be a helpful future perspective. As stated in this paper, the climate in Hiraizumi is not ideal for mummification, and though the mummies are cared for in a protected environment, there is the worry that slow, invisible changes might impact the condition of the mummies over time. In light of the recent declaration of Hiraizumi as a UNESCO World Heritage Site, an effort towards the future conservation of this important cultural heritage is highly advisable. A new investigation with modern, non-invasive technology may also answer the unsolved question of artificial or natural mummification, which might shed light on the unique cultural and religious practices of Hiraizumi in the 12^th^ century. The mummies of the Ōshū Fujiwara, together with the Konjiki-dō, remain unique artifacts bridging the complicated transition between ancient and medieval Japan and are, thus, of inestimable cultural, religious, and scientific significance.
